# The Role of D-Serine and D-Aspartate in the Pathogenesis and Therapy of Treatment-Resistant Schizophrenia

**DOI:** 10.3390/nu14235142

**Published:** 2022-12-02

**Authors:** Regina F. Nasyrova, Aiperi K. Khasanova, Kuanysh S. Altynbekov, Azat R. Asadullin, Ekaterina A. Markina, Arseny J. Gayduk, German A. Shipulin, Marina M. Petrova, Natalia A. Shnayder

**Affiliations:** 1 Institute of Personalized Psychiatry and Neurology, Shared Core Facilities, V.M. Bekhterev National Medical Research Centre for Psychiatry and Neurology, 192019 Saint Petersburg, Russia; 2 Department of Psychiatry, Russian Medical Academy for Continual Professional Education, 125993 Moscow, Russia; 3 International Centre for Education and Research in Neuropsychiatry, Samara State Medical University, 443016 Samara, Russia; 4 Republican Scientific and Practical Center of Mental Health, Almaty 050022, Kazakhstan; 5 Department of Psychiatry and Narcology, S.D. Asfendiarov Kazakh National Medical University, Almaty 050022, Kazakhstan; 6 Department of Psychiatry and Addiction, The Bashkir State Medical University, 450008 Ufa, Russia; 7 Centre for Strategic Planning and Management of Biomedical Health Risks Management, 119121 Moscow, Russia; 8 Shared Core Facilities “Molecular and Cell Technologies”, V.F. Voino-Yasenetsky Krasnoyarsk State Medical University, 660022 Krasnoyarsk, Russia

**Keywords:** D-serine, D-aspartate, disease-modifying therapy, treatment-resistant schizophrenia

## Abstract

Schizophrenia (Sch) is a severe and widespread mental disorder. Antipsychotics (APs) of the first and new generations as the first-line treatment of Sch are not effective in about a third of cases and are also unable to treat negative symptoms and cognitive deficits of schizophrenics. This explains the search for new therapeutic strategies for a disease-modifying therapy for treatment-resistant Sch (TRS). Biological compounds are of great interest to researchers and clinicians, among which D-Serine (D-Ser) and D-Aspartate (D-Asp) are among the promising ones. The Sch glutamate theory suggests that neurotransmission dysfunction caused by glutamate *N*-methyl-D-aspartate receptors (NMDARs) may represent a primary deficiency in this mental disorder and play an important role in the development of TRS. D-Ser and D-Asp are direct NMDAR agonists and may be involved in modulating the functional activity of dopaminergic neurons. This narrative review demonstrates both the biological role of D-Ser and D-Asp in the normal functioning of the central nervous system (CNS) and in the pathogenesis of Sch and TRS. Particular attention is paid to D-Ser and D-Asp as promising components of a nutritive disease-modifying therapy for TRS.

## 1. Introduction

Schizophrenia (Sch) is a severe mental disorder that affects approximately 0.5–1% of the population [1]. It includes positive, negative and cognitive symptoms and can lead to significant functional disorders and pronounced social maladaptation of patients [2]. There are several neurochemical hypotheses for the development of Sch: dopaminergic [3], kynurenic [4], glutamatergic [5] and others [6]. The leading hypothesis is dopaminergic, which has formed the basis of the approaches to Sch therapy. Antipsychotics (APs) are the first-line drugs for Sch treatment [7]. However, the issue of achieving a balance between the effectiveness and safety of APs remains open [8]. Other neurochemical hypotheses for the development of Sch continue to be explored as they may provide clues to discover a disease-modifying therapy for mental disorders [9].

**Figure 1:**
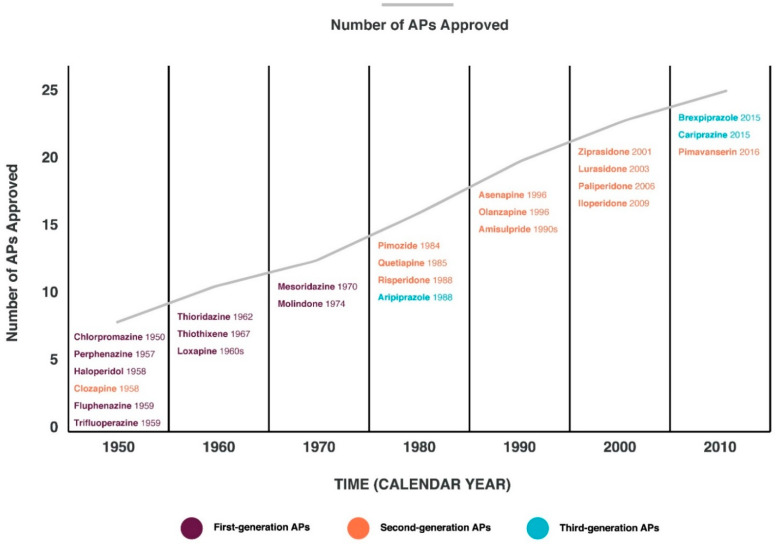
Timeline of antipsychotics (APs) approved by Food and Drug Administration (FDA) [9,11].

The response to APs of the first and new generations (Figure 1) [9] is variable, and it is still difficult to predict whether the therapy will be effective or not [10].

**Figure 2:**
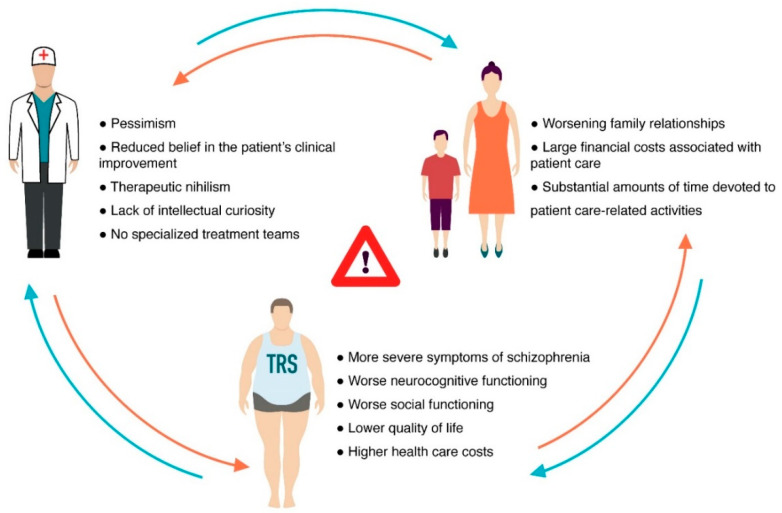
Burden of treatment-resistant schizophrenia (TRS) for the patient, family and the Healthcare System.

About 20–30% of patients with Sch do not have an adequate response ≥2 in terms of dose and duration of AP treatment [12]. This is the clinical definition of treatment-resistant Sch (TRS). As is known, TRS is a serious condition with associated clinical, social and medical expenses and consequences [13]. The definition of TRS has been revised several times. The first definition of TRS was proposed by Kane et al. [14]; this definition is based on the lack of response to Aps, using the example of clozapine. Most new definitions of TRS include failure to respond to at least two consecutive APs courses. In most cases, one of the two APs must be atypical, and have an adequate dose and duration of treatment (≥6 weeks). An adequate dose of an AP in the most recent report is defined as a daily dose equivalent to chlorpromazine ≥ 400 mg [15,16,17]. The absence of a therapeutic response to APs was indicated as a relative change in the evaluation scales (≥20% decrease in the scale of positive and negative symptoms of Sch) [17]. Thus, most patients with TRS may never achieve functional recovery [18], which leads to an increased burden of illness for the patient, family and the Healthcare System [19] (Figure 2).

Two types of TRS are known: (1) a type of resistance to APs that is already present at the onset of the disease; (2) the second type of resistance to APs, which develops later during the progression of Sch and/or after a period of successful therapeutic response to APs [20,21]. 

Treatment methods of Sch that include both typical (the first generation) and atypical (new generations) APs act primarily as brain dopaminergic receptor antagonists. Although these APs are highly effective in treating positive symptoms, their efficacy is limited in patients with persistent negative symptoms or cognitive impairment in patients with Sch [22]. Therefore, the development of a disease-modifying therapy for Sch [23], resistant to APs, is relevant. 

Associative genetic studies and genome-wide associative studies have identified over a hundred candidate genes as molecular biomarkers of TRS risk with modest effect [24,25,26]. Interestingly, some of these candidate genes have proteins involved in glutamatergic transmission, especially with the *N*-methyl-D-aspartate receptors (NMDARs) [27]. 

**Figure 3:**
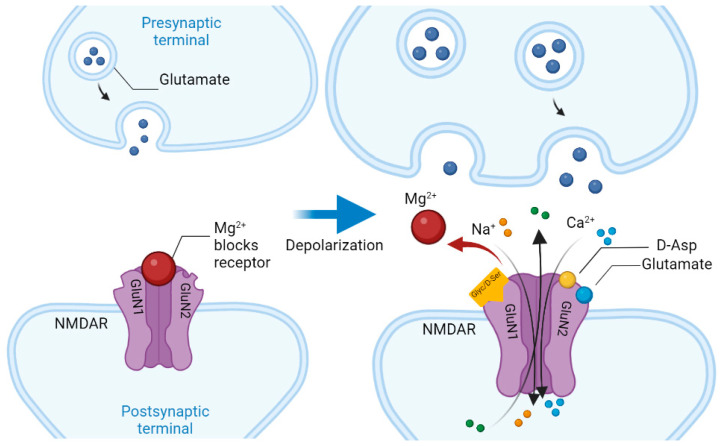
Scheme of the *N*-methyl-D-aspartate receptor (NMDR) functioning. Note: D-Asp—D-aspartate; D-Ser—D-serine; Glyc—glycine.

So, NMDARs are ligand-dependent ionotropic glutamate receptors, consisting of four subunits [28]. Three families of NMDAR subunits are known: GluN1, GluN2 (subtypes GluN2A, GluN2B, GluN2C and GluN2D) and GluN3 (subtypes GluN3A and GluN3B). At the same time, NMDAR is formed by two GluN1 subunits and either two GluN2 subunits, or a combination of GluN2 and GluN3 subunits, which form a channel (pore) [29]. GluN1 subunits have a glycine co-agonist or D-Serine (D-Ser) recognition sites, while GluN2 subunits have glutamate co-agonist recognition sites. In addition, NMDARs are at rest blocked by Mg^2+^ ions, which close the Ca^2+^ channel; NMDAR opens when three things happen simultaneously: (1) glutamate binds to its site on NMDAR; (2) glycine or D-Ser bind to their sites on NMDAR; and (3) depolarization of the neuron membrane occurs. At the same time, depolarization of the cell membrane removes Mg^2+^ ions from the channel (pore), and the binding of co-agonists Glyc and glutamate provides a voltage-dependent influx of Na^+^ and Ca^2+^ ions and outflow of K^+^ ions, causing postsynaptic effects of glutamate neurotransmission (Figure 3) [30]. 

NMDARs are located predominantly postsynaptically, but can also be located extrasynaptically. Activation of synaptic NMDARs usually promotes the survival of synapses and neurons, while excessive activation of extrasynaptic NMDARs by excess glutamate (“Glutamate shock”) can have a neurotoxic effect and cause neuronal death [31]. 

D-Aspartate (D-Asp) is a direct NMDAR agonist that excites both metabotropic glutamate receptors and dopaminergic neurons in the brain [32]. In addition, D-Asp is indirectly involved in the initiation and progression of neurodegenerative processes [33] and plays an important role in the neuroplasticity, physiology and morphology of neuronal dendrites, along with regulation of gray matter volume and brain activity [34,35]. 

**Figure 4:**
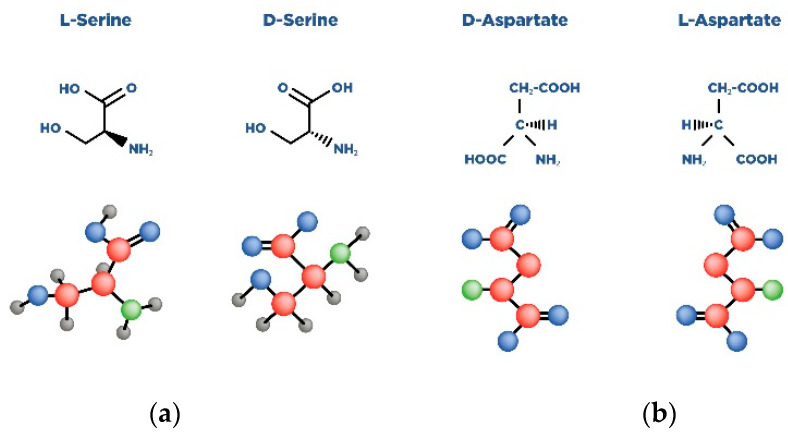
Scheme of L and D isomers of serine (**a**) and aspartate (**b**).

D-Ser and D-Asp are D-amino acids [36], and like most amino acids, they have a chiral carbon center, which allows the formation of two stereoisomers. These stereoisomers are mirror images of each other. Similar amino acids have both a left-handed (L) and a right-handed (D) enantiomer (Figure 4) [36].

L-amino acids are used in the human body as building blocks of proteins and intermediate products in biochemical processes [37]. 

Various studies in animal models and humans have demonstrated that D-amino acids, in particular D-Ser and D-Asp (Table 1) [38], are able to modulate various NMDAR-dependent processes, including synaptic plasticity, brain development, cognition and aging brain [39]. Dysfunctional NMDAR activity is associated with the etiology and pathophysiology of a wide range of psychiatric and neurological disorders, including Sch [40]. For example, according to one of the main hypotheses of Sch, glutamate activity in NMDARs is insufficient due to disturbances in the formation of glutamate NMDA synapses during brain development, while this hypothesis not only does not reject, but also confirms, the dopamine hypothesis of Sch [41]. 

This explains the growing number of studies on the role of D-Ser and D-Asp in the pathogenesis of Sch, TRS and disease-modifying therapy for TRS.

## 2. D-Aspartate

### 2.1. The Biological Role of D-Aspartate

D-Asp is an endogenous D-amino acid with the molecular formula C_4_H_7_NO_4_. Its average molecular weight is 133.1027 g/mol. D-Asp is located in the cytoplasm of neurons, extracellularly, and in peroxisomes. Laboratory methods detect D-Asp in the blood (21.0 ± 5.0 µM for male, 20.0 ± 5.0 µM for female), cerebrospinal fluid (0–1 µM), feces and urine (<1.13 µmol/mmol creatinine) [42]. 

Free D-Asp in the human body is determined in the central nervous system (CNS) and the human endocrine system [43]. Endogenous D-Asp is synthesized as a result of racemization of L-Asp in the CNS and endocrine tissue [44]; degradation of dietary protein; and microbial synthesis in the intestine [45]. 

Exogenous D-Asp enters the human body with food. It is found in vegetables (spinach, beets, iceberg lettuce, avocado, tomato and pepper), some mushrooms (shiitake, oyster mushrooms and chanterelles), fruits and berries (goji, black and red currants, gooseberries, elderberries, grapes and strawberries), some herbs (oregano, spearmint, peppermint, sweet basil, parsley, dill, watercress, rosemary, sorrel, dandelion and fenugreek), fish and nuts [42]. 

Nervous and endocrine tissues contain the enzymatic systems necessary to modulate D-Asp homeostasis, since they can synthesize and degrade this amino acid. Endogenous racemase activity D-Asp is involved in the biosynthesis of D-Asp from L-Asp, while D-Asp oxidase, a peroxisomal flavoprotein, metabolizes D-Asp to oxaloacetate, NH_3_ and H_2_O_2_ [46]. 

**Figure 5:**
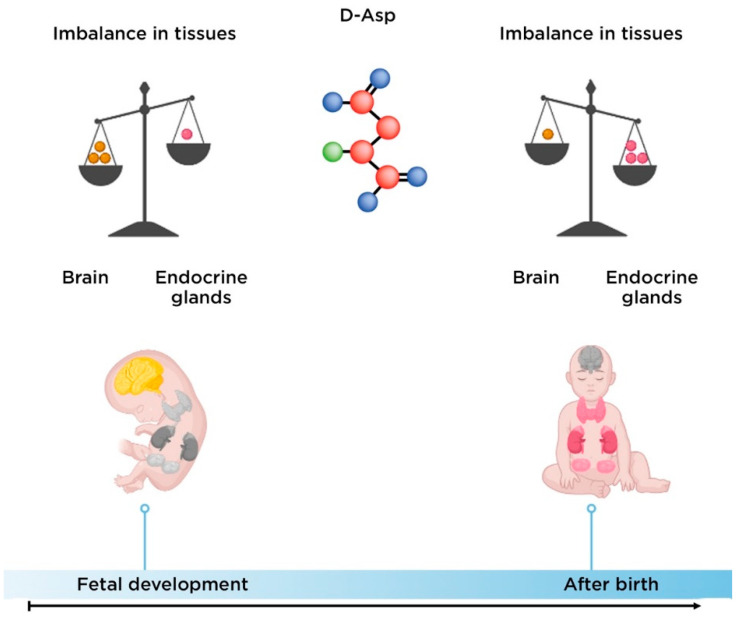
Change in D-aspartate balance between brain and endocrine tissue in the human ontogenesis.

The level of D-Asp in the CNS increases significantly during fetal development and decreases at birth. Conversely, the level of this amino acid in the endocrine tissue is low during the prenatal period and gradually increases after birth [47] (Figure 5).

Outside the CNS, the D-Asp function is known to regulate the synthesis and secretion of several hormones in endocrine and neuroendocrine tissues [48]. It induces the release of prolactin from the anterior pituitary gland, modulates the production of neurosteroids (oxytocin and vasopressin) in the posterior pituitary gland, and suppresses the secretion of melatonin in the pineal gland. In addition, D-Asp regulates the synthesis and release of testosterone by releasing gonadotropin-releasing hormone (GnRH) in the hypothalamus and luteinizing hormone (LH) in the pituitary gland [46]. In addition, D-Asp can promote animal and human reproduction, also directly activating the proliferation of spermatogonia and improving sperm quality [48]. 

Functionally, D-Asp corresponds to many, if not all, definitions of a classical neurotransmitter: (1) biosynthesis, degradation, uptake and release of the molecule occur in presynaptic neurons; and (2) D-Asp triggers a response in postsynaptic neurons after it is released from vesicles [49]. A high concentration of D-Asp was first detected in the brain of *Octopus vulgaris* Lam in 1977 [50]. Later, D-Asp was found in human nervous and endocrine tissues [51,52]. The level of D-Asp during ontogenesis has pronounced regional differences in the CNS, which suggests that D-Asp plays an important role in the development of the CNS. In the brain of rats at the stage of embryonic development, D-Asp is registered near the hindbrain and then spreads to the forebrain. D-Asp was first detected in the body of neurons of the outer layer of the nerve epithelium and then in axons as a distinct axonal layer was formed. These data support the notion that D-Asp is involved in the differentiation of brain neurons [53]. 

Intense D-Asp immunoreactivity was observed in the cortical plate and subventricular zone of the brain of rats in the early postnatal period. At the same time, D-Asp immunoreactivity decreases to an almost undetectable level in adult rats [54]. In all brain regions studied at all ages, D-Asp immunoreactivity was limited to neurons but not to glia. At the same time, immunohistochemical studies demonstrated D-Asp staining both in the bodies of neurons and in the pathways of neuronal migration. In addition, D-Asp is synthesized in hippocampal and prefrontal cortex neurons, which play an important role in the development of Sch [55]. 

D-Asp excites dopaminergic neurons and stimulates NMDARs and metabotropic glutamate receptors, preventing neuronal degeneration [32]. D-Asp oxidase regulates the homeostasis of the glutamatergic system when the level of D-Asp in the CNS changes and, thus, suppresses the initiation and progression of neurodegenerative processes [33]. 

D-Asp binds to the L-glutamate region of ionotropic NMDARs [56,57]. D-Asp in the mouse brain triggers intrinsic currents that counteract competitive and non-competitive NMDAR blockers, including D-2-amino-5-phosphonovaleric acid (D-AP5) and (+)-5-methyl-10,11-dihydro-5H-dibenzo(a,d)cycloheptene-5,10-imine maleate (MK801), ketamine and phencyclidine [58,59]. Residual D-Asp-dependent intrinsic currents persisted even after high doses of these NMDAR antagonists [58,60]. D-Asp also inhibits kainate-induced currents of the α-amino-3-hydroxy-5-methyl-4-isoxazolepropionic acid-type glutamate receptor (AMPAR) in hippocampal neurons and stimulates the metabotropic glutamate receptor 5 (mGluR5) [61,62,63]. 

D-Asp application triggers intrinsic currents in dopaminergic neurons in the hippocampus, striatum, spinal cord, and substantia nigra compacta by activating NMDAR and, to a lesser extent, AMPA and mGluR5 receptors [59,64]. 

Studies of primary cultures of neurons and synaptosomal drugs have demonstrated that D-Asp is stored in secretory organelles and released from axon terminals via vesicular exocytosis processes mediated by Ca^2+^ ions [65,66], or probably by spontaneous release or replacement carriers L-glutamate [67]. D-Asp efficiently crosses the blood–brain barrier (BBB) and is found in nanomolar concentrations in the extracellular space of the prefrontal cortex [63,68]. The level of D-Asp decreases in the CNS depending on the age of the person and is temporarily induced after high stimulation of the flow of K^+^ ions [69]. It is likely that intracellular D-Asp uptake may depend on the L-glutamate/L-Asp transport systems that recognize L-glutamate and both Asp enantiomers. In vitro studies have shown that D- and L-Asp are recognized and transported by glutamate transporter homolog from *Thermococcus kodakarensis* (Glt Tk) in the same manner and with comparable affinity [46]. 

To study the level of endogenous D-Asp, chromatographic methods are used in combination with the enzymatic cleavage of D-Asp. High-performance liquid chromatography (HPLC) and capillary electrophoresis (CE) are used to quantify D-Asp because they can separate chiral amino acids. This allows measurements of each enantiomer [70].

### 2.2. The Role of D-Aspartate in the Pathogenesis of Schizophrenia

The glutamate theory of Sch occurrence suggests that neurotransmission dysfunction caused by glutamate NMDARs may represent a primary deficit in this mental disorder and the development of TRS [71]. Decreased NMDARs function is considered a key change in the pathophysiology of TRS [10]. Two post-mortem studies have shown a significant decrease in D-Asp levels in prefrontal cortex neurons in Sch patients associated with increased D-Asp oxidase expression [16] or increased D-Asp oxidase enzymatic activity [72]. Sch patients showed altered NMDAR expression [73]. 

NMDAR agonists may enhance the therapeutic activity of APs in Sch patients, and glutamatergic agents improve negative symptoms of this disease [74].

Conversely, NMDAR antagonists may exacerbate Sch symptoms, and NMDARs hypofunction causes psychosis. NMDAR antagonists modulate dopaminergic activity in nucleus accumbens and different subregions of the prefrontal cortex and this modulation is related to positive symptoms, negative symptoms and cognitive deficits, respectively [75,76]. D-Asp has an effect on synaptic plasticity [34]; its condensate has been found in synaptic vesicles of axon terminals in the developing brain, further supporting the suggestion that D-Asp is an important neurotransmitter involved in CNS development [77]. 

A number of preclinical studies have demonstrated that D-Asp has an effect on several NMDAR-dependent phenotypes associated with Sch. The *D-Asp oxidase* gene knockout mice treated with D-Asp demonstrated that long-term elevation of D-Asp levels significantly reduced the deficit in neuronal pre-pulse inhibition induced by psychotomimetic drugs (amphetamine and MK-801) [78]. A single dose of phencyclidine, which simulates the symptoms of Sch in humans and animal models of Sch, reduced motor hyperactivity, ataxia and impairment of pre-pulse inhibition in D-Asp oxidase knockout mice. NMDAR antagonists impair social interaction in rodents an animal model reflecting social withdrawal [10,79]. Elevated D-Asp levels in D-Asp oxidase knockout mice counteract phencyclidine-induced dysfunctional activation of the cortico-limbic-thalamic pathways [79].

It was previously suggested that cortico-hippocampal disconnection in the CNS appears in Sch [80]. In this regard, an increased length of dendrites and a density of spinal cord neurons, as well as a greater functional cortical-hippocampal connectivity in the brains of *D-Asp oxidase* knockout mice were revealed [79]. An increased functional connection between the hippocampus and the cortex was also found in the rat brain after intragastric administration of D-Asp [81]. 

Errico et al. [82], in a study conducted on a small number of samples (n = 7) of brain tissue, revealed a decrease in the level of D-Asp (about 40%) in the postmortem prefrontal cortex of patients with chronic Sch. Also, a significant increase in the level of the messenger of ribonucleic acid (mRNA)—D-Asp oxidase was found in the same area of the brain [79]. Nuzzo et al. [83] in a study demonstrated on a larger number of samples (n = 20) recorded a statistically significant decrease in the level of D-Asp (about 30%) in the dorsolateral prefrontal cortex compared to the corresponding brain regions of apparently healthy people. The decrease in D-Asp in this region of the brain was associated with greater (about 25%) cortical activity of the D-Asp oxidase enzyme responsible for D-Asp catabolism.

### 2.3. D-Aspartate as a Promising Component of Disease-Modifying Therapy for Treatment-Resistant Schizophrenia

In addition to the neurochemical and functional studies described above, there have also been a few preclinical studies of D-Asp as a component disease-modifying therapy for Sch in animal models.

Errico et al. [78] conducted a study in an animal model with a knockout of the *D-Asp oxidase* gene. The mice aged 45 days to 4 months were orally administered D-Asp in solution (20 mM). The authors studied the therapeutic effect of D-Asp on animal behavior and electrophysiological processes in the CNS. With an increase in the level of D-Asp in the brain, NMDARs activity increased in vivo, the deficiency of prepulse inhibition caused by amphetamine and MK-801 decreased, and the cortical-striate long-term depression described in mice receiving long-term haloperidol (the first-generation AP) was prevented and increased NMDAR-dependent memory (modulation of hippocampal function). These results confirm the neuromodulatory effect of D-Asp in vivo on signaling through NMDARs and may open new therapeutic options for the correction of negative and positive symptoms of Sch and associated cognitive impairment in patients with TRS.

Sacchi et al. [84] demonstrated that modulation of D-Asp metabolism in the CNS may play an important role in modulating the therapeutic response to olanzapine (new-generation AP) in patients with TRS. Olanzapine, unlike other APs of the first and new generations, inhibits the enzymatic activity of D-Asp oxidase in vitro. In addition, long-term olanzapine administration can increase extracellular levels of D-Asp and L-glutamate in the prefrontal cortex of freely moving mice, but not in *D-Asp oxidase* knockout mice.

The use of D-Asp as a promising component of disease-modifying therapy for TRS is early on (Table 2), as studies are still being conducted in animal models.

## 3. D-Serine

### 3.1. Biological Role of D-Serine

D-Ser is an endogenous D-amino acid with the molecular formula C3H7NO3. Its average molecular weight is 105.0926 g/mol. D-Ser is located extracellularly in relation to the neuron. Laboratory methods were found in blood (2.28 ± 0.59 µM), saliva and urine (0.0021–10.1 µmol/mmol creatinine) [85]. 

Free D-Ser in the human body is determined in the CNS, adipose tissue, epidermis, intestines, kidneys, lungs, pancreas, placenta, platelets, fibroblasts, skeletal muscles, spleen and testicles [85]. Endogenous D-Ser is synthesized by serine racemase (SR), a pyridoxal-5′-phosphate (PLP)-dependent bifunctional type II fold enzyme, from L-Ser [86]. 

In humans, D-Ser is mainly cleaved by D-amino acid oxidase (DAO). The level of D-Ser in the tissues of the human body is tightly regulated by achieving a balance between its synthesis, degradation, absorption and/or transport. As with most amino acids, when a meal containing Ser is ingested, the molecule is extracted in the small intestine and absorbed into the bloodstream. It crosses the BBB, and enters neurons, where it is metabolized into glycine and many other molecules. Thus, the amount of Ser in cells is regulated by these metabolic processes. If too little is ingested, more Ser is converted from various sources. When too much is ingested, only a portion is converted to glycine, while the rest is metabolized to folate and many other proteins [86].

**Figure 6:**
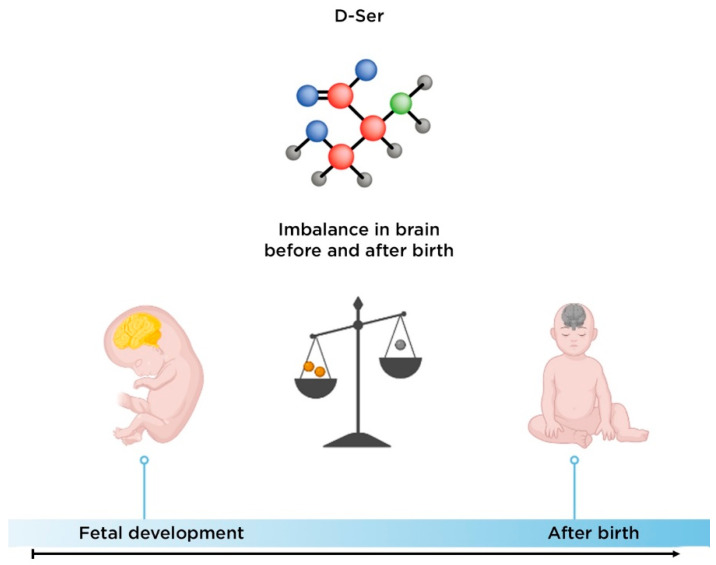
Change in D-serine in the human brain during ontogenesis. Notes: insufficiently studied in endocrine tissue.

The level of D-Ser in the CNS remains high during fetal development and postnatal life, even though its content in the brains of adolescents and aged individuals was about half that of fetuses [87] (Figure 6). We did not find data on changes in the level of this amino acid during ontogenesis in endocrine tissues. However, levels of D-serine in the endocrine system are much lower than those in the CNS, and the physiological role of D-serine in the endocrine systems remains unclear [43]. 

Exogenous D-Ser enters the human body with food. It is contained in berries (black raisin, green plum, wampee, goji, hawthorn, lantern fruit, cape gooseberry, mulberry, gooseberry, etc.), fruits (pitaya, lichee, mango, pineapple, guava, banana, coconut, apple and pear), beans, vegetables (olive, avocado, tomato and eggplant), etc. [85]. 

D-Ser is a potent NMDAR co-agonist, and its role in the brain is of great scientific and clinical interest. D-Ser is present in glia (mainly astrocytes) and CNS neurons. It is considered a glial transmitter [65] and as a neurotransmitter [88]. However, the role of glia and neurons in the therapeutic response to D-Ser is debatable [88]. Volosker et al. [88] suggested that astrocytes synthesize L-Ser, which is then transported to neurons for conversion into D-Ser.

NMDAR activation requires not only glutamate, but also a co-agonist. For many years, glycine was thought to be a co-agonist, and the site where it acts on NMDAR is called the glycine binding site. The regional distribution of D-Ser is more similar to that of NMDRs than that of glycine, and has a stronger affinity than glycine for the glycine binding site on the NR1 subunit of the NMDAR [71]. This indicates that D-Ser may be more important in neurotransmission via NMDARs.

The absence of D-Ser is one of the important factors in the reduction in long-term potentiation and cognition [89]. A decrease in D-Ser reduces the development of long-term potentiation, which is involved in memory consolidation [90]. Elevated D-Ser may improve recognition and memory [91].

The hypofunction of NMDARs has been described in patients with Sch and in an animal model of Sch; therefore, D-Ser is of great interest as the main co-agonist of NMDARs in the forebrain and hippocampus [92]. Glycine and D-Ser seem to act on different populations of NMDARs: D-Ser on synaptic receptors, and glycine on extrasynaptic receptors [32]. Probably, synaptic NMDARs have a neuroprotective effect, and extrasynaptic NMDARs can promote neurodegeneration [93]. An animal model of Sch shows that the basal activity of NMDARs, as well as the branching and density of spikes on neuronal dendrites, are reduced in mice with a knockout of the *SR* gene encoding SR, an enzyme that converts L-Ser to D-Ser. Long-term administration of D-Ser changed the expression of a protein associated with the neuronal cytoskeleton and caused a partial recovery of dendritic anomalies in the same model [49]. Fujita et al. [94] reported that young and adolescent rodents that were prenatally exposed to the activation of maternal immunity showed reduced expression of NMDAR subunits of hippocampal neurons, which led to the initiation of cognitive impairment in adulthood. In addition, Fujita et al. [94] demonstrated that the addition of D-Ser to drinking water led to a reduction in cognitive deficits in an animal model of Sch. Spatial and reverse memory deficits in Sprague-Dawley rats treated with the NMDAR antagonist phencyclidine can be eliminated by the administration of D-Ser [95].

### 3.2. The Role of D-Serine in the Development of Schizophrenia

Preclinical studies in an animal model of Sch have demonstrated that a decrease in the level of D-Ser in the CNS due to a decrease in SR activity can cause Sch symptoms, including stereotypy, cognitive impairment, impaired prepulse inhibition (a measure of sensorimotor gating), persistent latent inhibition (a measure of inhibition of learning and cognitive flexibility) and a lack of social interaction [38,96,97]. 

A decrease in D-Ser levels in cerebrospinal fluid and peripheral blood has been reported in patients with Sch [98]. MacKay et al. [96] reported that the level of D-Ser in the blood of patients with Sch is lower than in the control group, which has also been shown by other researchers [99]. In addition, blood levels of D-Ser and the ratio of D-/L-Ser enantiomers were significantly increased in Sch patients with a good therapeutic response to clozapine (second-generation AP) [68].

Basal D-Ser levels are reduced in Sch patients, potentially due to genetic variation in the genes encoding SR and DAO, which are involved in D-Ser synthesis and degradation, respectively [71]. Significant reductions in D-Ser levels have been found in cerebrospinal fluid in naïve patients with Sch [99]. Balu et al. [100] treated mice with a knockout of the *SR* gene with D-Ser 300 mg/kg day 1, 150 mg/kg days 2–21 subcutaneously. Restoration of neuroplasticity, increase in BDNF protein expression and reversing the freezing deficit were demonstrated. 

Decreased activation of the NMDAR co-agonist site may be seen in TRS, and an increase in D-Ser may reduce the risk and severity of TRS by reversing NMDAR dysfunction [96].

It has been hypothesized that D-Ser may prevent the development of TRS. Pathological and anatomical studies revealed anomalies in the expression of enzymes that modulate D-Ser. Thus, pathoanatomical studies have demonstrated an increase in the expression and activity of DAO in the cerebellum of patients with Sch [93]. Patients with Sch also had higher levels of DAO mRNA in hippocampal neurons [101] and increased DAO activity in cortical neurons [102]. The clinical significance of high levels of DAO in the cerebellum in patients with Sch and TRS is not yet known. However, cerebellar dysfunction in patients with Sch may manifest as mild neurological symptoms or impaired motor function and cognition [103].

Anomalous SR expression in hippocampal and cerebral cortex neurons in patients with Sch [104] has been shown. Although the results of other authors were contradictory and indicated a local increase in the level of SR [61], measures of SR activity in the brains of patients with Sch may be informative in determining whether D-Ser synthesis is altered in this psychiatric disorder.

In general, neurochemical and pathological studies (Table 3) indicate that abnormal levels of D-Ser in the CNS and peripheral blood may be present in patients with TRS. These results support further exploration of the role of D-Ser as a component of TRS disease-modifying therapy.

### 3.3. D-Serine as a Promising Component of Disease-Modifying Therapy for Treatment-Resistant Schizophrenia 

A 4-week, open-label, randomized clinical trial demonstrated that D-Ser at high doses (>30 mg/kg/day) as a disease-modifying therapy in addition to AP resulted in positive, negative and general Positive and Negative Syndrome Scale (PANSS) symptoms improvement and reduction in cognitive dysfunction in patients with Sch and schizoaffective disorders [65]. 

In most studies, D-Ser was administered at a dose of 30 mg/kg (~2000 mg/day) with a significant but small positive effect on positive and negative symptoms of Sch, including improvement in cognitive function [105]. A high dose (60 mg/kg/day, ~4000 mg/day) may be even more effective than a lower dose, with significant therapeutic effects on cognition [65]. In patients with a high risk of developing Sch, D-Ser statistically significantly reduced the severity of negative symptoms of Sch according to the Scale of Prodromal Symptoms [88]. Despite the negative results of a multicenter study of D-Ser at a dose of 30 mg/kg [64], a meta-analysis including studies with higher doses of D-Ser demonstrated a significant clinical effect on negative symptoms of Sch [60,71]. 

In subsequent studies of D-Ser [71,88], including a double-blind, placebo-controlled, randomized, parallel-groups negative symptoms clinical trial, individuals at risk for Sch were given this amino acid at a dose of 60 mg/kg/day for 16 days. It has been demonstrated that D-Ser may be useful for the treatment of prodromal symptoms of Sch. Another double-blind, placebo-controlled, randomized clinical trial showed that D-Ser at a dose of 60 mg/kg/day significantly improved mismatch negativity (MMN), a neurophysiological biomarker of NMDARs activity, in patients with Sch [71]. Ermilov et al. [106] compared high doses (3000 mg/day) of D-Ser with high doses of olanzapine (30 mg/day) in patients with TRS and concluded that D-Ser is effective as a monotherapy. Interestingly, a very high dose of D-Ser up to 4000 mg/day did not cause adverse drug reactions (ADRs) [92]. 

In several clinical studies, D-Ser has been used as a disease-modifying therapy for Sch at various doses (30–120 mg/kg/day) in addition (co-administration) to APs. When prescribing D-Ser with non-clozapine APs (APs of new generations, except clozapine), there was a reduction in negative and positive symptoms of Sch, as well as an improvement in cognitive function in these patients [96]. In addition, even when administered at very high doses (120 mg/kg/day), D-Ser did not have serious ADRs. A double-blind, placebo-controlled, randomized clinical trial by Heresco-Levy et al. [107] studied the effect of the additional administration of D-Ser to patients taking olanzapine or risperidone. At the end of the 6-week treatment, patients treated with D-Ser showed an improvement in cognitive function, a decrease in positive and negative symptoms of Sch, and a significant reduction in comorbid depressive disorders. The simultaneous measurement of the concentration of amino acids in the serum did not reveal any fluctuations in other amino acids, except for an increase in the serum level of D-Ser.

A meta-analysis of clinical studies comparing therapy with glycine modulatory site agonists, including D-Ser, showed that all of these NMDAR agonists significantly reduced Sch symptoms, including improved cognitive function and reduced affective symptoms, when D-Ser was added to new generation APs, with the exception of clozapine [56,59].

However, some studies have obtained conflicting results. For example, some authors [64,108,109] reported negative results of disease-modifying therapy with the addition of D-Ser. In addition, there have been conflicting results regarding the therapeutic effect of D-Ser (30 mg/kg/day) on Sch-negative symptoms and Sch-associated cognitive impairment. However, high-dose D-Ser (>60 mg/kg/day) has shown a statistically significant improvement in Sch patients in most studies. High doses of D-Ser may be required to potentiate the NMDAR-mediated activation of dopaminergic receptors, as well as to achieve therapeutic serum D-Ser levels and the subsequent predicted increase in brain concentrations of this amino acid.

As previously described, the addition of D-Ser to clozapine did not increase the therapeutic efficacy of this second-generation AP [59]. This can be explained by the fact that clozapine promotes the release of D-Ser and glutamate [58] and may have agonist or partial agonist activity of NMDARs [6,51,52]. It has been suggested that older Sch patients taking clozapine do not respond to D-Ser, but this issue has not been well studied [59].

One problem with Sch disease-modifying therapy with D-Ser is the fact that it is rapidly metabolized by DAO in the human body [110]. This can lead to a decrease in the bioavailability of exogenous D-Ser and requires the use of high doses. However, this strategy of disease-modifying therapy increases the risk of developing neurotoxic ADRs (primarily peripheral neuropathy) and nephrotoxicity [96]. Although, in a number of clinical studies of high and very high doses of D-Ser (120 mg/kg/day for 4 weeks), serious ADRs, including nephrotoxicity, have not been registered [65]. Rare cases of transient microproteinuria without glucosuria and without changes in serum creatinine levels are possible, which quickly disappeared after discontinuation of D-Ser [88].

In a 16-week intervention study conducted by the same group of authors [88], patients at risk of Sch were given D-Ser at a dose of 60 mg/kg/day. At the same time, two clinical cases of D-Ser withdrawal due to abnormal indicators of kidney function associated with D-Ser were registered. At the same time, long-term D-Ser-induced ADRs after a 16-week treatment period are not well understood.

Summary results of clinical trials of D-Ser as a new therapeutic strategy for TRS are presented in Table 4.

## 4. Discussion

Sch is one of the most common mental disorders that affects the working population in many countries; although, incidence rates range from 0.2% in some African countries (Central African Republic, Somalia) to 0.5% in the USA, Australia and New Zealand [111]. Although some of the differences in these epidemiological rates may be due to different approaches and timing of the diagnosis of this mental disorder, differences in the prevalence of Sch can also be explained by studies that demonstrate that the lack of certain nutrients in the diet can contribute to the development of Sch as an additional externally modifiable risk factor [112,113]. Notably, some authors have shown that deficiencies in essential vitamins, minerals and polyunsaturated fatty acids are often reported in the population, but rarely in patients with Sch [114,115,116]. Other authors demonstrate that the daily supplementation of essential nutrients is often effective in reducing Sch symptoms and may be considered an adjunctive (disease-modifying) therapy strategy for TRS. Free-form amino acids are one of the most effective and safest nutrients available to support mental health, not only are they the building blocks of proteins that provide structure to the CNS, but they are also critical to the proper functioning of the CNS, as some amino acids are key to maintaining adequate levels of neurotransmitters (including dopamine, norepinephrine, serotonin, etc.), as demonstrated in this review using D-Asp and D-Ser). Although most Sch patients consume adequate amounts of protein, they may still be deficient in amino acids, the causes of this phenomenon are usually associated with impaired digestion and intestinal microbiota [117,118], when how much protein the patient consumes with food is of less importance, since his body does not can effectively break down exogenous protein into individual amino acids and their deficiency occurs. When this happens, recommending more complex proteins is not the answer to amino acid deficiencies. It is known that the consumption of free-form amino acids does not require additional digestion as amino acids are readily available and absorbed directly into the systemic circulation; this provides easy access to the amino acids necessary for the functioning of the CNS. For many Sch patients, amino acid supplementation may be one of the most consistently effective disease-modifying therapies, improving the expected therapeutic effect on APs.

Amino acid supplements can also reduce the positive and negative symptoms of Sch because they are either converted to neurotransmitters or have effects similar to neurotransmitters in the CNS, as demonstrated by D-Ser and D-Asp. The mechanisms underlying the therapeutic effects of D-Asp and D-Ser are variable and continue to be studied. However, their modulating effect on glutamatergic and dopaminergic neurotransmission is beyond doubt. At the same time, D-Ser as a component of TRS disease-modifying therapy has been more studied than D-Asp. However, studies in animal models of Sch, TRS and ultra-TRS (UTRS) patients demonstrate better prognosis (better outcomes in catamnesis) and a lower risk of developing ADRs when exogenous D-Asp and D-Ser are given as dietary supplements. In addition, diet therapy, long forgotten because it is not of interest to pharmaceutical companies, is also important in providing nutritional support to patients with TRS. In a number of countries, there is huge resistance to the use of supplements as a disease-modifying therapy for Sch and TRS, in particular from psychiatrists, mainly due to their lack of knowledge on this issue. Most psychiatrists prefer to use prescription APs and other medications (e.g., antidepressants, mood stabilizers, etc.) that have passed randomized controlled trials and are registered by the FDA. 

However, the use of first and new-generation APs does not solve the problem of TRS in more than 30% of cases and sometimes leads to the development of serious ADRs, especially in chronic psychopharmacotherapy [9]. Thus, if psychiatrists avoid such TRS disease-modifying therapy due to a lack of knowledge or willingness to use new Sch treatments that are not supported by pharmaceutical companies and not yet approved by the FDA, they may jeopardize patient recovery due to their own laziness or selfishness [118]. Timely and correct medical diagnosis of TRS and a clear understanding of all possible therapeutic strategies should always be the prerogative of the practicing psychiatrist in the treatment of mental disorders. At the same time, it takes some time for clinicians to become familiar with all available TRS disease-modifying therapy options, including nutritional support (particularly exogenous D-Ser and D-Asp) and dietary advice that includes foods rich in these amino acids [85] (Table 5). However, this is an important task of modern psychiatry, which is undesirable to ignore. 

The authors believe that psychiatrists who treat patients with Sch should be aware of available nutritional support methods, at appropriate doses, and possible ADRs. Such an approach is important in providing alternative and adjuvant (disease-modifying) therapy to their patients. At the same time, any form of treatment, including the administration of exogenous D-Asp and D-Ser and foods rich in these amino acids, should be monitored, and doses and duration of treatment should be adjusted individually for each patient as necessary to achieve optimal results.

**Figure 7:**
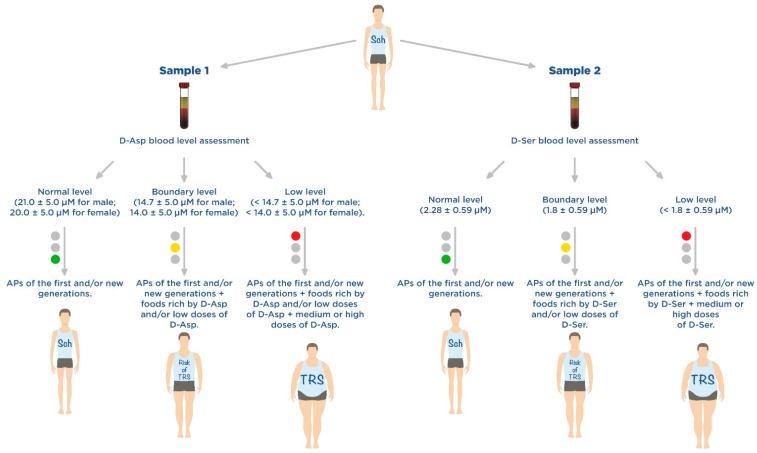
Personalized nutritional support algorithm for patients with schizophrenia (Sch) and treatment-resistant schizophrenia (TRS): the choice of diet and/or disease-modifying therapy for Sch depends on the level of D-aspartate (D-Asp) and/or D-serine (D-Ser) in the blood and personal risk of developing TRS-associated with a deficiency of these D-amino acids; green—low risk of TRS; yellow—medium risk of TRS; red—high risk of TRS.

So, our summarized results of fundamental and clinical studies of D-Asp and D-Ser demonstrated that low blood levels of these amino acids can be considered additional metabolic biomarkers of TRS, so their study may be useful in patients at risk of developing TRS. At the same time, low serum levels of D-Asp and/or D-Ser may indicate that this group of patients with Sch requires dietary adjustment (diet therapy) and/or additional administration of supplements containing these amino acids (Figure 7). Undoubtedly, large randomized intervention studies are needed to study in detail the effect of various doses of D-Asp and D-Ser (low, medium, high and very high) in patients with risk of Sch, TRS and UTRS, but even now it can be recognized that these amino acids have a positive effect on positive and negative symptoms of Sch and cognitive impairments. 

## 5. Conclusions

In recent years, there has been an increase in scientific and clinical interest in the role of D-Asp and D-Ser in the treatment of various mental disorders. This growth in basic and clinical research is partly due to our evolving understanding of the neurobiological basis of Sch and TRS, which implies the use of certain nutrients as a promising disease-modifying therapy for a variety of reasons. It is important for practicing psychiatrists to increase their knowledge of new nutritional support strategies as a component of a disease-modifying therapy for Sch that modulates and enhances the therapeutic response to first and new-generation APs, including the use of D-Asp and D-Ser, which are not yet evidence based. Future research on the role of D-Asp and D-Ser should focus on determining which Sch patients may benefit most from diets and supplements containing these amino acids to further elucidate their underlying mechanisms of action, including in reducing the risk of developing TRS and its therapy.

## Figures and Tables

**Table 1:** Functions of D-aspartate and D-serine in the central nervous system.

Amino Acid	Receptor	Function
D-aspartate	Synaptic NMDAR	Binds to the L-Glut region of ionotropic NMDARs.
D-serine	Synaptic NMDAR	A full agonist of the NMDAR Glyc modulatory site. Physiological ligand of the co-agonist site. Reduces NMDAR-mediated transmission in vitro via non-vesicular release via ASC-1 transporters.

Note: ASC-1—alanine-serine-cysteine-1; Glyc—glycine; L-Glut—L-glutamate; NMDAR—*N*-methyl-D-aspartate receptor.

**Table 2:** Preclinical studies of D-aspartate as a new therapeutic strategy for treatment-resistant schizophrenia.

Authors, Year	Country	Study Design	Results
Errico et al. [78]	Italy	Prospective study. Mice with a knockout of the *DDO* gene. The dose of D-Asp is 20 mM per os.	Increase in NMDARs activity. Decrease in pre-pulse inhibition deficit. Prevention of long-term cortical-striate depression. Enhancement of NMDAR-dependent memory.
Sacchi et al. [84]	Italy	Prospective study. Mice with a knockout of the *DDO* gene. The dose of D-Asp is 20 mM D-Asp per os. The dose of olanzapine is 5 mg/kg intraperitoneal.	Modulation of the therapeutic response to APs via D-Asp. Inhibition of the enzymatic activity of DDO. Increase in D-Asp and L-Glut extracellular level in the prefrontal cortex via olanzapine.

Note: APs—antipsychotics; D-Asp—D-aspartate; DDO—D-Asp oxidase; L-Glut—L-Glutamate; NMDAR—*N*-methyl-D-aspartate receptor.

**Table 3:** Preclinical studies of D-serine as a new therapeutic strategy for treatment-resistant schizophrenia.

Authors, Year	Country	Study Design	Results
MacKay et al. [96]	Canada	Prospective study. Mice with a mutation in the *SR* gene. Biochemical markers, mRNA expression, behavioral phenotypes and pharmacological responses were examined.	Appearance of Sch symptoms: stereotypy, cognitive impairment, impaired prepulse inhibition, lack of social interaction.
Balu et al. [100]	USA	Prospective study. Mice with a knockout of the *SR* gene. The dose of D-Ser is 300 mg/kg day 1, 150 mg/kg days 2–21 subcutaneously.	Restoration of neuroplasticity. Increase in BDNF protein expression. Reversing the freezing deficit.

Note: BDNF—brain-derived neurotrophic factor; D-Ser—D-serine; SR—serine racemase; Sch—schizophrenia.

**Table 4:** Clinical studies of D-serine as a new therapeutic strategy for treatment-resistant schizophrenia.

Authors, Year	Country	Study Design	Results
Kantrowitz et al. [65]	USA	Open prospective study. 42 adults with SSDS. The dose of D-Ser: 30, 60 or 120 mg/kg/day in addition to APs (the first and new generations).	Positive, negative and general PANSS symptoms improvement, reduction in neurocognitive dysfunction.
Pei et al. [105]	Taiwan	Open placebo-controlled prospective studies; 37 adults with SSDS. The dose of D-Ser: 1500–4000 mg/day in addition to APs (the first and new generations).	MMN and cortical plasticity improvement. Weak decrease in the severity of negative and positive Sch symptoms. Improvement of negative symptoms at doses above 3600 mg/day of D-Ser.
Kantrowitz et al. [88]	USA	Pilot, double-blind, placebo-controlled randomized parallel-group study. 20 patients aged between 13 and 35 years with clinical high risk of Sch. The dose of D-Ser: 60 mg/kg.	Improvement of negative symptoms.
Weiser et al. [64]	Israel	Multicenter, double-blind, randomized, placebo-controlled study; 97 adults with SSDS. The dose of D-Ser: 2000 mg/day in addition to APs (the first and new generations).	No significant difference between drug and placebo.
Tsai et al. [60]	USA and Taiwan	Meta-analysis; 102 adults with SSDS. The dose of D-Ser: 2000 mg/day in addition to APs (the first and new generations).	Reduction in Sch symptoms, improvement of cognitive functions and reduction in affective symptoms.
Kantrowitz et al. [71]	USA	Double-blind, placebo-controlled study; 21 adults with SSDS. The dose of D-Ser: 60 mg/kg/day.	MMN improvement. Reduction in PANSS overall scores.
Ermilov et al. [106]	Israel	Pilot, double-blind, placebo-controlled study; 10 adults with SSDS. The dose of D-Ser: 3000 mg/day.	Significant reduction in PANSS positive symptom scores and overall PANSS score.
MacKay et al. [96]	Canada	Double-blind, placebo-controlled studies, open-label studies. Different number of adults with SSDS. The dose of D-Ser: 30–120 mg/kg/day alone or in addition to APs (non-clozapine APs).	Reduction in negative and positive symptoms of Sch. Improvement of cognitive functions.
Heresco-Levy et al. [107]	USA	Double-blind, placebo-controlled study; 39 adults with SSDS. The dose of D-Ser: 30 mg/kg/day in addition to APs (olanzapine, risperidone).	Improvement of cognitive functions, reduction in positive and negative symptoms of Sch and reduction in comorbid depressive disorders.
Singh et al. [56]	UK	Meta-analysis; 99 adults with SSDS. The dose of D-Ser: 2000 mg/day in addition to APs (the first and new generations).	Reduction in Sch symptoms, improvement of cognitive functions and reduction in affective symptoms.
Iwata et al. [108]	Canada	Meta-analysis; 175 adults with SSDS. The dose of D-Ser—30 mg/kg/day in addition to APs (the first and new generations).	No significant difference between drug and placebo.
Tsai et al. [59]	Taiwan	Double-blind study; 10 adults with SSDS. The dose of D-Ser: 30 mg/kg/day in addition to APs (clozapine).	No increase in therapeutic efficacy of clozapine.

Note: APs—antipsychotics; D-Ser—D-serine; SSDS—schizophrenia spectrum disorders; Sch—schizophrenia; MMN—mismatch negativity; PANSS—Positive and Negative Syndrome Scale.

**Table 5:** Foods rich in D-aspartate and D-serine.

D-aspartate	D-serine
**Berries**	
Green plum	Black raisin
Goji	Chinese plum
Blackcurrant	Green plum
Redcurrant	Wampee
Gooseberry	Goji
Vaccinium (Blueberry, Cranberry, Huckleberry)	Monk fruit
Bilberry	Hawthorn
Lingonberry	Lantern fruit
Elderberry	Cape gooseberry
Grape	Mulberry
Loganberry	Black mulberry
Strawberry guava	Black chokeberry
Strawberry	Blackcurrant
	Redcurrant
	Gooseberry
	Cloudberry
	Red raspberry
	Black raspberry
	Black elderberry
	Rowanberry
	Vaccinium (Blueberry, Cranberry, Huckleberry)
	Lowbush blueberry
	Sparkleberry
	Highbush blueberry
	American cranberry
	Bilberry
	Lingonberry
	Muscadine grape
	Common grape
**Beans**	
Lima bean	Cannellini bean
Common bean	Scarlet bean
Adzuki bean	Lima bean
Mung bean	Common bean
Hyacinth bean	Broad bean
Winged bean	Adzuki bean
	Gram bean
	Mung bean
	Climbing bean
	Hyacinth bean
	Moth bean
	Winged bean
	Bean
	Yellow wax bean
	Green bean
**Fruits**	
Lichee	Pitaya
Mango	Lichee
Passion fruit	Mango
Pineapple	Passion fruit
Guava	Pineapple
Pomegranate	Custard apple
Tamarind	Guava
Banana	Pomegranate
Longan	Tamarind
Star fruit	Banana
Cherimoya	Longan
Coconut	Rambutan
Jackfruit	Star fruit
Kumquat	Abiyuch
Papaya	Acerola
Common persimmon	Breadfruit
French plantain	Natal plum
Prickly pear	Cherimoya
Sapodilla	Coconut
Mamey sapote	Durian
Kiwi	Jackfruit
Feijoa	Java plum
Persimmon	Kumquat
Apple	Mammee apple
Pear	Purple mangosteen
	Papaya
	Common persimmon
	Pitanga
	Plains prickly pear
	French plantain
	Prickly pear
	Malabar plum
	Sapodilla
	Mamey sapote
	Soursop
	Sugar apple
	Kiwi
	Feijoa
	Persimmon
**Mushrooms**	
Common mushroom	Jew’s ear
Shiitake	Common mushroom
Oyster mushroom	Shiitake
Maitake	Enokitake
Chanterelle	Oyster mushroom
	Cloud ear fungus
	Maitake
	Chanterelle
	Morchella (Morel)
**Vegetables**	
Spinach	Iceberg lettuce
Swiss chard	Pea shoots
Common beet	Water spinach
Jute	Spinach
Endive	Chicory leaves
Lettuce	Common beet
Garden cress	Corn salad
Fruit vegetables	Jute
Olive	Malabar spinach
Avocado	Rocket salad
Garden tomato	Swamp cabbage
Eggplant	Garden cress
Groundcherry	Radish
Pepper	Burdock
Parsnip	Celeriac
Radish	Carrot
Burdock	Potato
Celeriac	Asparagus
Carrot	Onion
Rape	
Carrot	
Garden rhubarb	
Celery stalks	
**Aquatic foods**	
Fishes	Fishes
Seaweed	Seaweed
Crustaceans	Mollusks
Mollusks	
Roe	
**Meat**	
Cattle (Beef, Veal)	Cattle (Beef, Veal)
Chicken	Chicken
Pork	Pork
**Coffee**	
**Eggs**	
**Milk products**	

## Data Availability

Not applicable.
